# Climatic Stress during Stand Development Alters the Sign and Magnitude of Age-Related Growth Responses in a Subtropical Mountain Pine

**DOI:** 10.1371/journal.pone.0126581

**Published:** 2015-05-14

**Authors:** Paloma Ruiz-Benito, Jaime Madrigal-González, Sarah Young, Pierre Mercatoris, Liam Cavin, Tsurng-Juhn Huang, Jan-Chang Chen, Alistair S. Jump

**Affiliations:** 1 Biological and Environmental Sciences, School of Natural Sciences, University of Stirling, Stirling, United Kingdom; 2 Forest Ecology and Restoration Group, Department of Life Sciences, University of Alcala, Alcalá de Henares, Madrid, Spain; 3 School of Medicine and Research Center for Biodiversity, China Medical University, Taichung, Taiwan; 4 Department of Forestry, National Pingtung University of Science and Technology, Nei Pu Hsiang, Pingtung, Taiwan; Chinese Academy of Sciences, CHINA

## Abstract

The modification of typical age-related growth by environmental changes is poorly understood, In part because there is a lack of consensus at individual tree level regarding age-dependent growth responses to climate warming as stands develop. To increase our current understanding about how multiple drivers of environmental change can modify growth responses as trees age we used tree ring data of a mountain subtropical pine species along an altitudinal gradient covering more than 2,200 m of altitude. We applied mixed-linear models to determine how absolute and relative age-dependent growth varies depending on stand development; and to quantify the relative importance of tree age and climate on individual tree growth responses. Tree age was the most important factor for tree growth in models parameterised using data from all forest developmental stages. Contrastingly, the relationship found between tree age and growth became non-significant in models parameterised using data corresponding to mature stages. These results suggest that although absolute tree growth can continuously increase along tree size when trees reach maturity age had no effect on growth. Tree growth was strongly reduced under increased annual temperature, leading to more constant age-related growth responses. Furthermore, young trees were the most sensitive to reductions in relative growth rates, but absolute growth was strongly reduced under increased temperature in old trees. Our results help to reconcile previous contrasting findings of age-related growth responses at the individual tree level, suggesting that the sign and magnitude of age-related growth responses vary with stand development. The different responses found to climate for absolute and relative growth rates suggest that young trees are particularly vulnerable under warming climate, but reduced absolute growth in old trees could alter the species’ potential as a carbon sink in the future.

## Introduction

Forests are key ecosystems for the global carbon cycle [1] and provide multiple ecosystem services fundamental to human well-being [2]. During the last century a rapid increase in forest growth has been observed worldwide alongside elevated atmospheric carbon dioxide (e.g. [3, 4]). However, signs of forest response saturation to CO_2_ increases have already been reported [5] and there is increasing evidence that rising atmospheric CO_2_ can no longer offset the negative impacts of warming on tree growth (e.g. [6]). Understanding the variation in the contribution of individual trees to stand productivity is particularly important because although large trees may have a disproportionate role in accumulating biomass (e.g. [7]) differential sensitivity to climate has been reported as trees age (e.g. [8, 9]). Consequently, we urgently need to understand how interactions between age and climate warming may affect individual tree growth to adequately predict stand-level responses and likely future impacts on the carbon cycle e.g. through absolute tree growth, [1, 7] and vulnerability to climate warming (e.g. through relative tree growth, [10]).

Absolute and relative age-related growth responses have been widely studied at stand level, because absolute growth informs about net changes in biomass while relative growth depends on the previous size. Absolute age-related growth generally shows hump-shaped responses as stand develops (e.g. [11, 12]). Declines in absolute stand growth at mature stages have been related to tree-level physiological constrains, as reduced carbon gains and photosynthetic efficiency (i.e. due to unbalanced carbon gains and respiration); nutrient decreases and hydraulic constraints (i.e. less supply of water and nutrients in large trees); and changes in root-to-shoot allocation (e.g. [13, 14]). At tree level, relative growth rates generally decrease with age and/or size [15] but contrasting absolute growth responses have been recently reported and discussed (see S1 Table): from no evidence of age-related responses [16] and hump-shaped responses (e.g. [17, 18]), to a continuous biomass increase with tree size and age (e.g. [7, 19]). The continuous increase in absolute tree growth with age as opposed to the traditional hump-shaped growth at stand level could be due to changes through stand development, for example: (i) physiological adjustments such as crown optimization (i.e. leaves are organised in mature stages to maximize carbon gains) and increased leaf packing (i.e. leaf area index can increase with age producing higher growth, [20] can occur; and (ii) differential resource availability, stand heterogeneity and species dominance [21, 22]. Furthermore, growth could be largely reduced under increased climatic stress and high competition levels [23, 24]. However, it is not completely understood if interactions between climate and age as stands develop might lead to different age- and size-dependent absolute and relative growth patterns (e.g. [25]).

Climate-growth relationships with annual resolution can be constructed from tree ring information accounting for species-specific idiosyncrasies and individual tree responses to climate [26–28]. There is increasing evidence of a negative effect of climate warming in forests from low latitudes not limited by low temperatures [29, 30]. Contrasting sensitivity of absolute growth to climate depending on age and/or size has been reported, from no-effect and higher sensitivity to climate in old and/or large trees (e.g. [9, 31]) to a higher sensitivity of young and/or small trees (e.g. [32]) or no age- and/or size-effect (e.g. [33]). On the one hand, the higher sensitivity to climate in old and/or large trees have been linked to high hydraulic stress or decreased carbon gains under high temperatures (see e.g. [15, 31]). Larger effects of climate on old and big trees may particularly impact the global carbon cycle, because large trees have a disproportional role on forest carbon cycle (e.g. [7]). On the other hand, larger effects of climate on small and/or young trees have been linked to fast responses to climate conditions and longer growing seasons, which may lead to changes in xylem formation and growth (e.g. [32, 34]). Larger sensitivities to climate in young as opposed to old trees may imply bottlenecks in forest persistence under warming climate (e.g. [10]).

The study of the effects of climate warming on tropical forests has been particularly scarce and controversial compared to higher latitudes [30, 35, 36]. In this study, we examined both absolute and relative tree level growth responses to age and climate of a sub-tropical pine species, *Pinus taiwanensis* Hayata, along a large altitudinal gradient of *c*. 2,200 vertical meters. We used tree ring data to account for age-dependent growth responses during the 20^th^ century at the tree level while considering climatic variability (see [37, 38]). The large altitudinal and temporal gradient covered by this study provides a good example a mountain sub-tropical species where increased temperature may have altered age-related growth patterns at the tree level during stand development. Our main objectives are: (i) to determine how age-related growth responses vary when forests are assessed from developing stages to maturity, and (ii) to quantify the relative importance and interactions between tree age and climate on absolute and relative age-related tree growth responses. We addressed three main hypotheses: (i) absolute growth would increase and relative growth decrease as trees age when all stand developmental stages are considered together, but slight declines or no-effects would be observed in mature stages; (ii) increased temperature would cause reduced tree growth, particularly in trees located in the warmest sites covered by this study; and (iii) young trees would be more responsive to climate in relative growth terms, whereas we do not have a clear expectation for absolute growth. The results provided here constitute new evidence to better understand how age-related growth patterns depend on stand development and predict potential impacts of warming climate depending on tree ontogeny.

## Material and Methods

### Study Site and Analysed Variables

**Fig 1:**
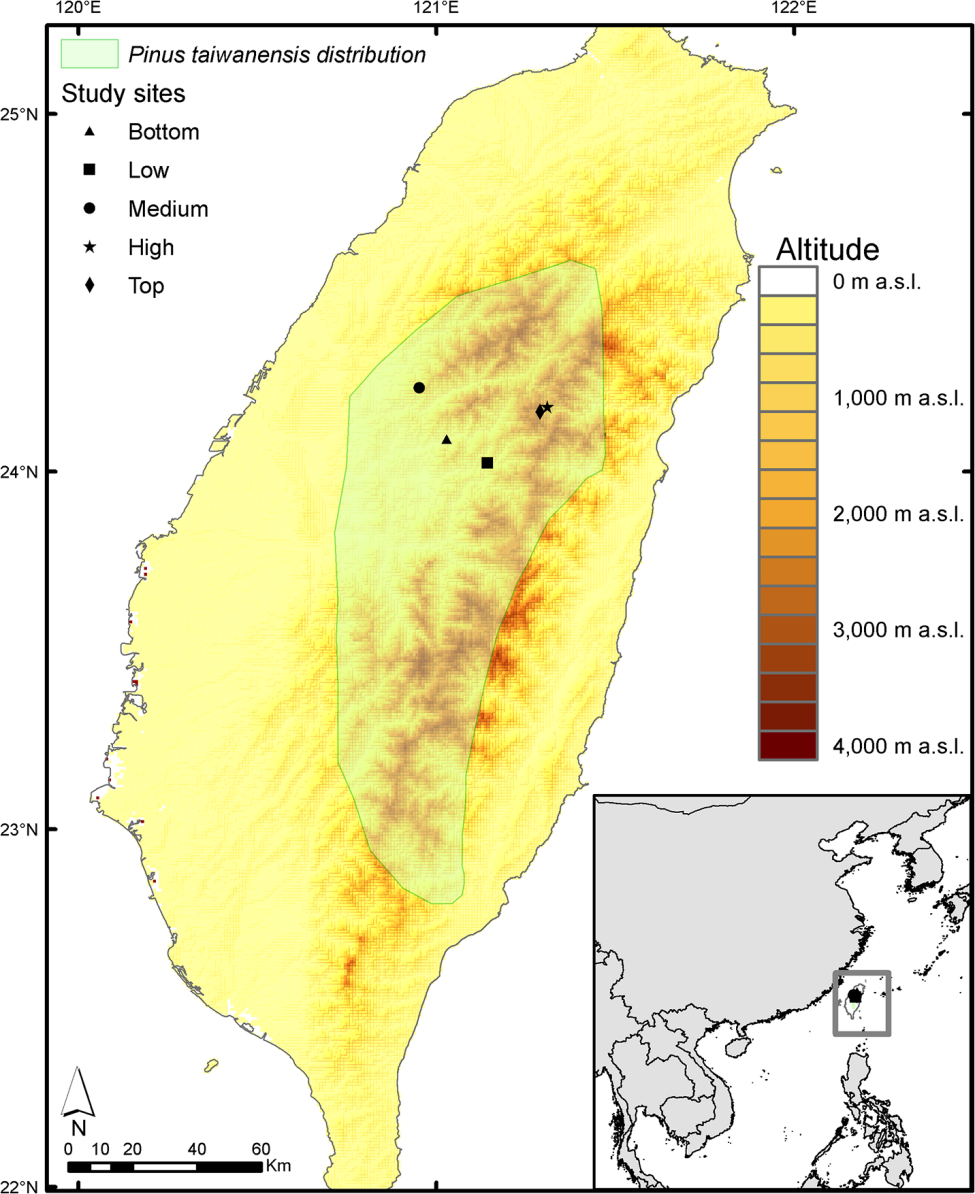
Map of *Pinus taiwanensis* sites sampled in Taiwan. We included *Pinus taiwanensis* distribution [65] and the altitudinal gradient in Taiwan (digital elevation model STRM30, SRTM V2, http://www2.jpl.nasa.gov/srtm/).

*Pinus taiwanensis* is the most widely distributed pine species in Taiwan, occurring from warm temperate to sub-alpine climates. *P*. *taiwanensis* is an early successional species and can occur as pure stands throughout its altitudinal gradient as well as mixed stands with broadleaved species to isolated trees in high-elevation montane meadows. However, stands of this species can also constitute a successional end-point under severe environmental conditions, such as on precipices or shallow and stony soils (e.g. [39]). The sites selected in this study covered a large altitudinal gradient which varied markedly on species composition and richness (see Fig 1).

**Fig 2:**
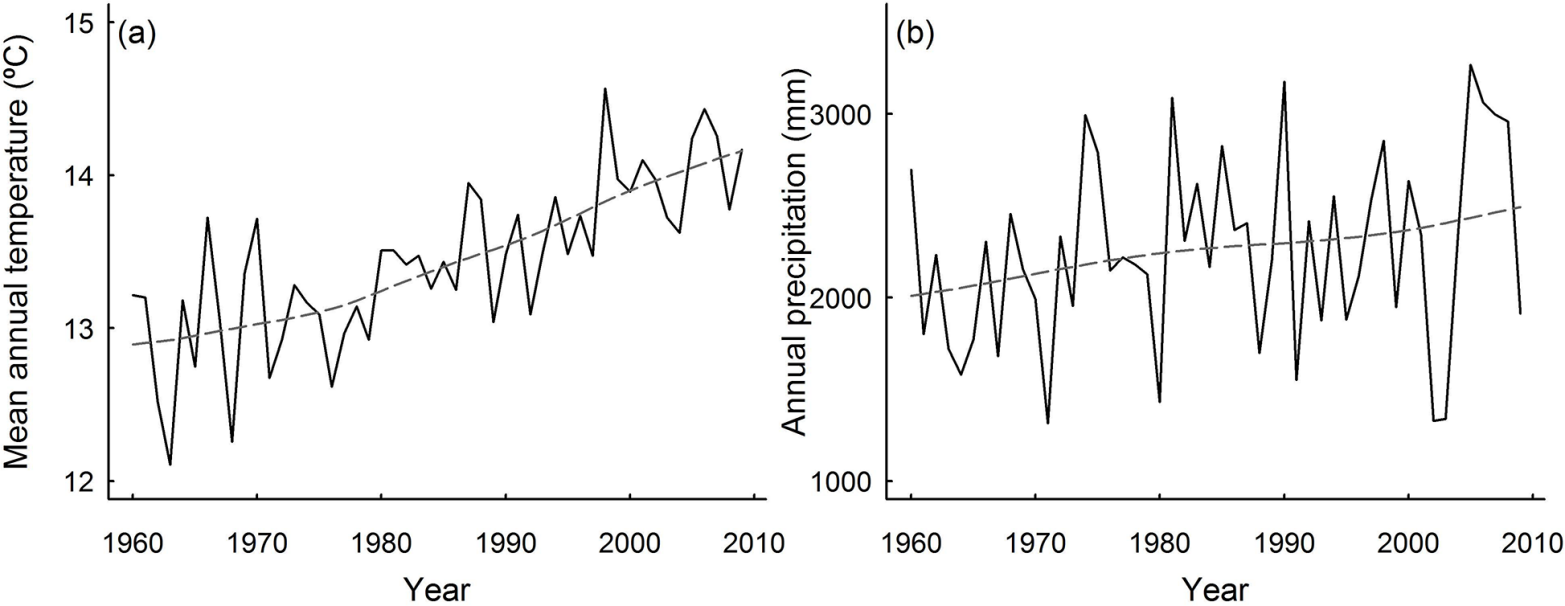
Temporal change in climate from 1960 to 2009 for the study area. We show the mean climate in all the sites sampled for the period 1960–2009 of (a) mean annual temperature (°C) and (b) annual precipitation (mm).

*Pinus taiwanensis* grows within the subtropical biome in central Taiwan covering a large altitudinal gradient. *Pinus taiwanensis* dominates during early stages of stand development, remaining a dominant vegetation component in small patches across the large altitudinal gradient covered in this study (2,250 m a.s.l.). Five study sites were established covering the altitudinal distribution of *P*. *taiwanensis* forests (from 695 to 2,945 m a.s.l.). Sites covered a gradient of forest composition, from species rich sub-tropical lowland forests to relatively species poor high altitude forest surrounded by the montane conifer, *Abies kawakamii* (see S2 Table). For each of the five study sites, monthly mean annual temperature (°C) and annual precipitation (mm) was obtained from 1960 to 2009 (see Fig 2). Mean annual temperature (°C) for each site was interpolated from the records of Alishan meteorological station (2,413 m a.s.l.) according to the regional altitudinal temperature lapse of -0.5°C each 100 m [40]. Annual precipitation (mm) was obtained from interpolated precipitation data provided by Taiwan from the Central Weather Bureau, Taiwan.

Tree core samples were collected during 2010 in the five study sites (see Table 1). We did not required specific permission to perform the field sampling because the sites were not located in private lands or protected areas. Furthermore, our field studies did not involve manipulation of endangered or protected species. From each study site, 20 dominant or co-dominant trees were selected and two or three cores were collected from each individual tree using a 4.3 mm increment borer at breast height (1.30 m). Samples were prepared for tree-ring analysis using standard dendroecological techniques and scanned at 3,200 d.p.i. using a flatbed scanner and saved as. jpg files. Total ring width was measured to an accuracy of 0.001 mm using CooRecorder v.2.3.13 [41]. A small number of cores that were not readable were excluded. In order to detect dating and measurements errors, ring-width series were checked with COFECHA v606P software [42]. Sections of any core that showed a poor match with the COFECHA master series for each site (i.e. correlation < 0.3) were identified. Where poor matching of correctly dated segments resulted from twisted, compressed or decayed wood, these cores were excluded from the analysis. Ring width for each year was averaged between the cores taken from each tree to produce a final ring width series for each individual. Statistics of ring width chronologies (see S3 Table) shows that mean sensitivity ranged between 0.170 and 0.414 (i.e. range of easy dating, [34]).

**Table 1:** Summary characteristics of the study sites along *Pinus taiwanensis* distribution.

	Bottom	Low	Medium	High	Top
Altitude (m a.s.l.)	695	1,213	2,166	2,444	2,941
Mean annual temperature (°C)	19.42	16.83	12.06	10.67	8.19
Annual precipitation (mm)	2,195	1,905	2,136	2,353	2,678
Latitude	121° 1’ 47” E	121° 08’ 39” E	120° 57’ 11” E	121° 18’ 38” E	121° 17’ 23” E
Longitude	24° 5’ 25” N	24° 01’ 26” N	24° 14’ 3” N	24° 10’ 48” N	24° 10’ 2” N
Species richness [Woody] (No. species)	36 [27]	29 [20]	23 [18]	11 [7]	5 [3]
No. trees [No. cores]	19 [37]	19 [35]	13 [24]	20 [39]	20 [38]

Ring width values (mm yr^-1^) were used to estimate the age at breast height. After determination of the full core width (mm), a central area of the tree cross-section remained with unknown age. This area was divided by average ring width for the first recorded 10 years of the tree growth to estimate the number of years of this section. This estimated value was added to the number of years of growth recorded for the core to provide an approximate measure of absolute tree age (No. years) in each of the five study sites when the samples were collected in 2010.

Ring width (*RW*, mm yr^-1^) was converted to tree basal area increment (*BAI*, mm^2^ yr^-1^) using dplR library [43] in R version 3.0.1 [44], according to the following standard formula:

$$BAI\;=\;\pi \;\left(R_{n}^{2}\;-\;R_{n-1}^{2}\right)$$ (1)

Where *R* is the radius of the tree (mm) and *n* is the year of the tree ring formation. Finally, we also calculated relative tree growth (*RTG*, % yr^-1^), as the annual basal area increment with respect to the basal area of the previous year. Relative growth rate was also selected because it is easily comparable among different tree and stand developmental stages [45].

### Statistical Analysis

**Fig 3:**
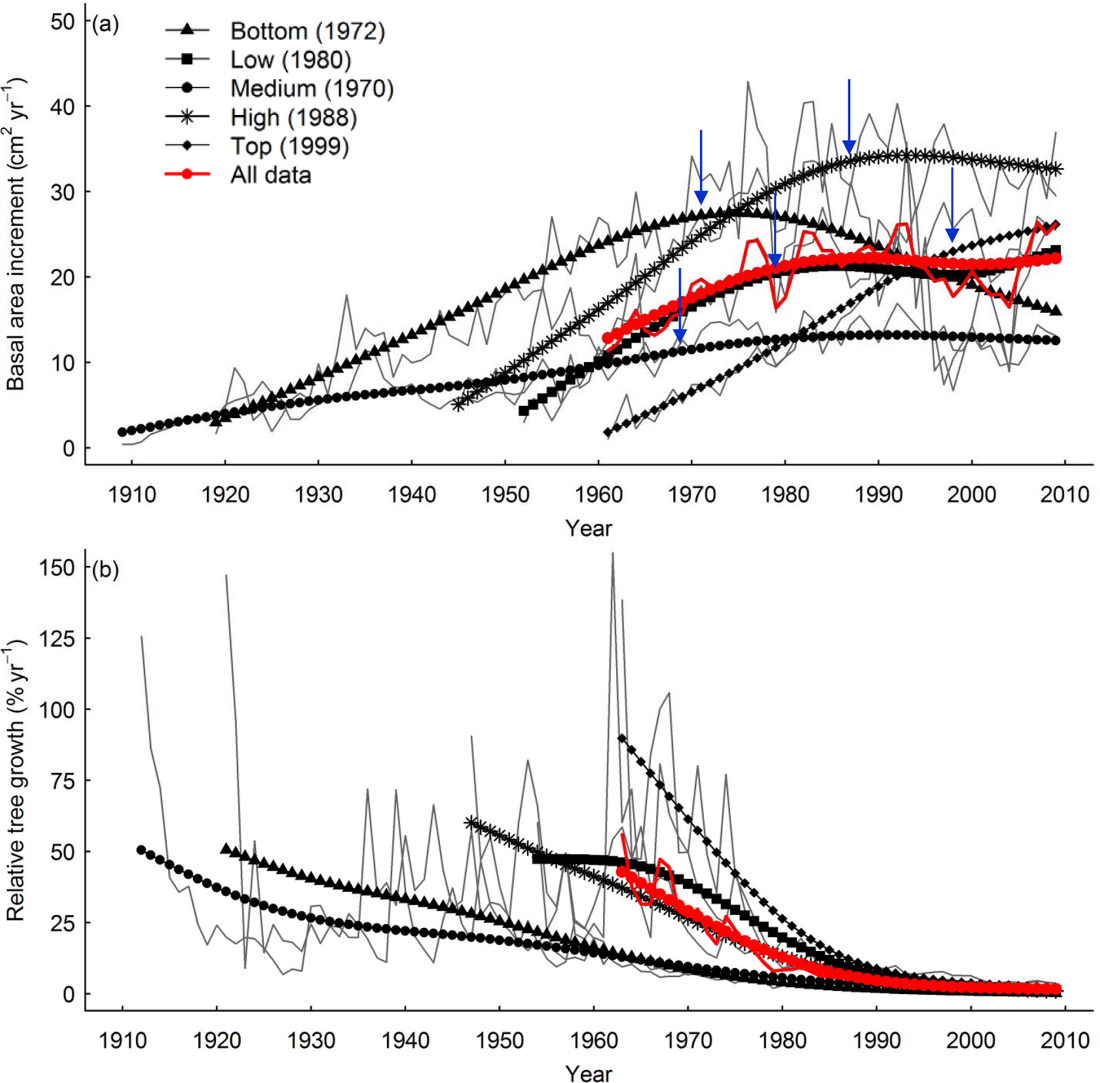
Basal area increment and relative growth from 1909 to 2009 in each site ((a) and (b), respectively). Arrows show the inflection point for each site and the legend gives the year following the inflection point for each site indicating the beginning of the mature phase of growth.

We modelled basal area increment (*BAI*, mm^2^ yr^-1^) and relative tree growth (*RTG*, %) using linear mixed-effects models for two data-sets: (i) data covering all developmental stages (i.e. using data from 1960 to 2009 where climatic information was available), and (ii) mature-stage data following stabilization of basal area increment as tree age increases (i.e. using data from the inflection point for each site showed in Fig 3). To split the data in the mature development stage, mean basal area increment for each site and all data were smoothed using a cubic smoothing spline and smoothing parameters that varied between 0.8 and 0.9 and obtaining the same results in R.3.0.1 [44], allowing us to highlight growth trends while retaining their variability. We calculated an inflection point of basal area increment, marking the start point at which the mature phase occurs in each site from the next year of the inflection point to avoid the growth peak during stand development (see Fig 3). All statistical analysis were performed using *BAI* and *RGT* data for each tree and year (i.e. smoothed data was not used).

Linear mixed-effects models were fitted using a normal distribution of residuals and an identity link for the response variable (using log(*BAI*) or log(*RTG*) as response variable). The linear mixed-effects models had a normal error distribution and an identity link. For the two sets of models we included one fixed predictor of tree age (*TA*, No. years): (i) tree age in models parameterised using all developmental stage data (i.e. this measure varies within time); and (ii) absolute tree age in models parameterised with mature stage data (i.e. absolute tree age). We also included two fixed predictor climatic variables: mean annual temperature (*MAT*, °C), and annual precipitation (*PP*, mm; see mean values in Table 1). Based on our initial hypotheses and preliminary analysis of response variables along explanatory predictors (see S1 and S2 Figs), we tried differential functional forms, including linear or nonlinear terms for each explanatory variable and the pair-wise interactions *TA* × *MAT* and *TA* × *PP* (see S4 and S5 Tables). All the numerical predictor variables were standardised (i.e. the mean was subtracted from each value and divided by the standard deviation), enabling the interactions to be tested and compared [46]. Tree identity nested in site identity was included in the model as a random effect to account for non-independence due to their similar localities. Additionally, in order to detect co-linearity between explanatory variables, we calculated the variance inflation factors (VIFs) for each predictor variable. VIFs calculate the degree to which co-linearity inflates the estimated regression coefficients as compared with the orthogonal predictors. Our results confirmed that co-linearity was not a major problem in our data (VIF < 1.5).

The most parsimonious model was determined using AIC (Akaike Information Criterion) as an indicator of both parsimony and likelihood, where a difference lower than 10 indicated no support for the most complex model [47]. To identify the best-supported model we constructed all possible combinations of alternative models, from the maximal model considering both the main effects and the pair-wise interactions between the fixed effects. However, as we were interested in analysing the effect of tree age on basal area increment and relative tree growth, we always retained tree age as a variable in order to compare its effect between different models. Therefore, tree age was retained even when it was not supported by the most parsimonious model for comparative purposes (see S5 Table). Repeated analyses with tree age excluded showed that parameters estimates were not affected by its inclusion in the model (data not shown). From the final models selected, each variable and interaction term was dropped, using the differences in AIC to quantify the relative importance of each predictor variable.

Parameter estimation and confidence intervals of the selected models were obtained using restricted maximum likelihood (REML), which minimizes the likelihood of the residuals from the fixed-effect portions of the model [46]. The parameter estimates provide the basis for determining the magnitude of the effect of a given process, with maximum likelihood estimates of parameter values close to zero indicating no effect. We calculated confidence intervals from the posterior distribution of parameter estimates using the bootstrapping methods available in the lme4 package. Marginal pseudo-*R* ^*2*^ (proportion of variance explained by fixed factors alone) and conditional pseudo-*R* ^*2*^ (proportion of variance explained by both the fixed and random factors) were used to provide an estimation of variance explained by fixed and random terms [48]. All analyses were performed in R version 3.0.1 [44], using the “lme4” package [49].

## Results

### Absolute and Relative Tree Growth in all Developmental Stages and Mature Forests

Using data from all developmental stages, the best model of basal area increment (*BAI*) included all main effects and the pair-wise interaction between tree age and mean annual temperature (see Table 2), according to the following form:

$$\text{log}\left(BAI\right)\;=\;\beta_{1}+\beta_{2}\left(TA\right)+\beta_{3}\left(TA^{2}\right)+\beta_{4}\left(MAT\right)+\beta_{5}\left(MAT^{2}\right)+\beta_{6}\left(PP\right)+\;\beta_{7}\left(TA\right)\left(MAT\right)$$ (2)

where β_1_ to β_7_ are the estimated parameters and the predictor variables were: tree age (*TA*), mean annual temperature (*MAT*) and annual precipitation (*PP*).

**Table 2:** Alternative models of basal area increment and relative tree growth based on Akaike Information Criterion.

Basal area increment (mm^2^ yr^-1^)			Relative tree growth (% yr^-1^)		
All developmental stages	AIC	∆AIC	All developmental stages	AIC	∆AIC
***Full***	***6551*.*91***	***0*.*00***	***Full***	***6639*.*04***	***0*.*00***
No PP	6575.02	23.11	No PP	6668.82	29.77
No TA	6868.41	316.50	No MAT	6669.83	30.79
No MAT	7049.07	497.15	No TA	9735.89	3096.84
No interaction TA × MAT	6765.97	214.06	No interaction TA × MAT	6654.46	15.42
			No interaction TA × PP	6666.64	27.60
Basal area increment (mm^2^ yr^-1^)			Relative tree growth (% yr^-1^)		
Mature stage data	AIC	∆AIC	Mature stage data	AIC	∆AIC
*No TA*	*3920*.*38*	*0*.*00*	*No TA*	*4453*.*84*	*0*.*00*
**Full**	**3928.15**	**7.76**	**Full**	**4458.11**	**4.27**
No PP	3943.981	23.60	No PP	4496.841	43.00
No MAT	4108.489	188.11	No MAT	5111.643	657.80

Full models include the main effects of tree age (TA), mean annual temperature (MAT) and annual precipitation (PP). Alternative models ignore the effects (‘No’) of the main effects of each explanatory variable or interactions. The best fitting model is determined by ∆AIC value of zero and it is given in italics. The selected model includes the effect of tree age and it is given in bold.

Additionally, in relative tree growth (*RTG*) models an interaction between tree age (*TA*) and annual precipitation (*PP*) was supported by the best model. Therefore, the best model of relative tree growth (*RTG*) using data from all developmental stages followed the next form:

$$\text{log}\left(RTG\right)\;=\;\beta_{1}+\beta_{2}\left(TA\right)+\beta_{3}\left(TA^{2}\right)+\beta_{4}\left(MAT\right)+\beta_{5}\left(MAT^{2}\right)+\beta_{6}\left(PP\right)+\;\beta_{7}\left(TA\right)\left(MAT\right)\;+\beta_{8}\left(TA\right)\left(PP\right)$$ (3)

where β_1_ to β_8_ are the estimated parameters. Marginal pseudo-*R* ^*2*^ of the *BAI* and *RTG* models varied between 0.25 and 0.70 (i.e. variance explained by the fixed terms), and conditional pseudo-*R* ^*2*^ varied between 0.74 and 0.89 (i.e. variance explained by the fixed and random terms, see Table 3 for the estimated parameter values within each model and response variables and S3 and S4 Figs for residuals).

**Table 3:** Parameters of the final models of basal area increment and relative tree growth.

**Basal area increment (mm** ^**2**^ **yr** ^**-1**^ **), all developmental stages:** R_m_ = 0.2510, R_c_ = 0.7458 (see Eq 2)						**Relative tree growth (% yr** ^**-1**^ **), all developmental stages:** R_m_ = 0.7033, R_c_ = 0.8946 (see Eq 3)					
**Parameters**	Variables*	**Estimates**	**SE**	**LCI**	**UCI**	**Parameters**	Variables*	**Estimates**	**SE**	**LCI**	**UCI**
β_1_	(*Intercept*)	7.576	0.277	7.034	8.119	β_1_	(*Intercept*)	1.3414	0.2213	0.9076	1.7752
β_2_	*TA*	0.128	0.019	0.091	0.165	β_2_	*TA*	-1.2663	0.0190	-1.3035	-1.2292
β_3_	*TA* ^2^	-0.031	0.009	-0.049	-0.014	β_3_	*TA* ^2^	0.2457	0.0090	0.2281	0.2633
β_4_	*MAT*	-0.387	0.096	-0.575	-0.198	β_4_	*MAT*	-0.3488	0.0941	-0.5333	-0.1643
β_5_	*MAT* ^2^	-0.034	0.053	-0.138	0.069	β_5_	*MAT* ^2^	0.0323	0.0533	-0.0723	0.1368
β_6_	*PP*	0.052	0.009	0.034	0.070	β_6_	*PP*	0.0277	0.0092	0.0096	0.0458
β_7_	*TA* × *MAT*	-0.314	0.021	-0.355	-0.274	β_7_	*TA* × *MAT*	-0.1034	0.0211	-0.1448	-0.0621
						β_8_	*TA* × *PP*	0.0541	0.0090	0.0365	0.0718
**Basal area increment (mm** ^**2**^ **yr** ^**-1**^ **), mature stages:** R_m_ = 0.4268, R_c_ = 0.9208. (see Eq 4)						**Relative tree growth (% yr** ^**-1**^ **), mature stages:** R_m_ = 0.4666, R_c_ = 0.9842 (see Eq 4)					
**Parameters**	Variables*	**Estimates**	**SE**	**LCI**	**UCI**	**Parameters**	Variables*	**Estimates**	**SE**	**LCI**	**UCI**
β_1_	(*Intercept*)	7.563	0.554	6.476	8.649	β_1_	(*Intercept*)	0.3298	1.4906	-2.5918	3.2514
β_2_	*TA*	-0.006	0.075	-0.152	0.140	β_2_	*TA*	-0.1186	0.0628	-0.2416	0.0045
β_3_	*MAT*	-1.199	0.101	-1.397	-1.001	β_3_	*MAT*	-3.1473	0.1157	-3.3740	-2.9207
β_4_	*MAT* ^2^	-0.375	0.052	-0.478	-0.273	β_4_	*MAT* ^2^	-0.2947	0.0593	-0.4109	-0.1785
β_5_	*PP*	0.058	0.012	0.035	0.081	β_5_	*PP*	0.0917	0.0134	0.0655	0.1179

Parameters and variables related to the parameters (Parameters and Variables, respectively), mean parameter estimates (Estimates), standard error (SE), 95% confidence intervals (LCI and UCI), marginal pseudo-R^2^ (R_m_) and conditional pseudo-R^2^ (R_c_) for basal area increment (mm^2^ yr^-1^) and relative tree growth (% yr^-1^) models performed with all data and mature stage data. Variables acronyms: *TA*, tree age (No. years); *MAT*, mean annual temperature (°C); *PP*, annual precipitation (mm).

Using data from mature stages of growth the best models of basal area increment (*BAI*) and relative tree growth (*RTG*) only included the effects of climatic variables (see S5 Table). However, in order to compare individual tree growth responses with models parameterised using data from all developmental stages, we included the main effects of the predictor variables explored, according to the following form:

$$\text{log}\left(BAI,RTG\right)\;=\;\beta_{1}+\beta_{2}\left(TA\right)+\beta_{3}\left(\text{MAT}\right)+\beta_{4}\left(MAT^{2}\right)+\beta_{5}\left(\text{PP}\right)$$ (4)

where β_1_ to β_5_ are the estimated parameters and the predictor variables were: tree age (*TA*), mean annual temperature (*MAT*) and annual precipitation (*PP*). Marginal pseudo-*R* ^*2*^ of the models varied between 0.42 and 0.43 (i.e. variance explained by the fixed terms), and conditional pseudo-*R* ^*2*^ varied between 0.92 and 0.98 (i.e. variance explained by the fixed and random terms, see Table 3 for the estimated parameter values within each model and response variables and S3 and S4 Figs for residuals).

### Effects of Tree Age and Climate on Absolute and Relative Tree Growth

AIC model comparisons indicate that in the models considering all developmental stages, tree age (*TA*) and mean annual temperature (*MAT*) were the most important determinants of individual tree growth (Table 2). Annual precipitation was generally less important, although retained by the best model (Table 2). Contrastingly, for models parameterised using data from the mature stage of growth, the model comparison suggests that tree age is not supported by the best models (see **∆**AIC in Table 2).

**Fig 4:**
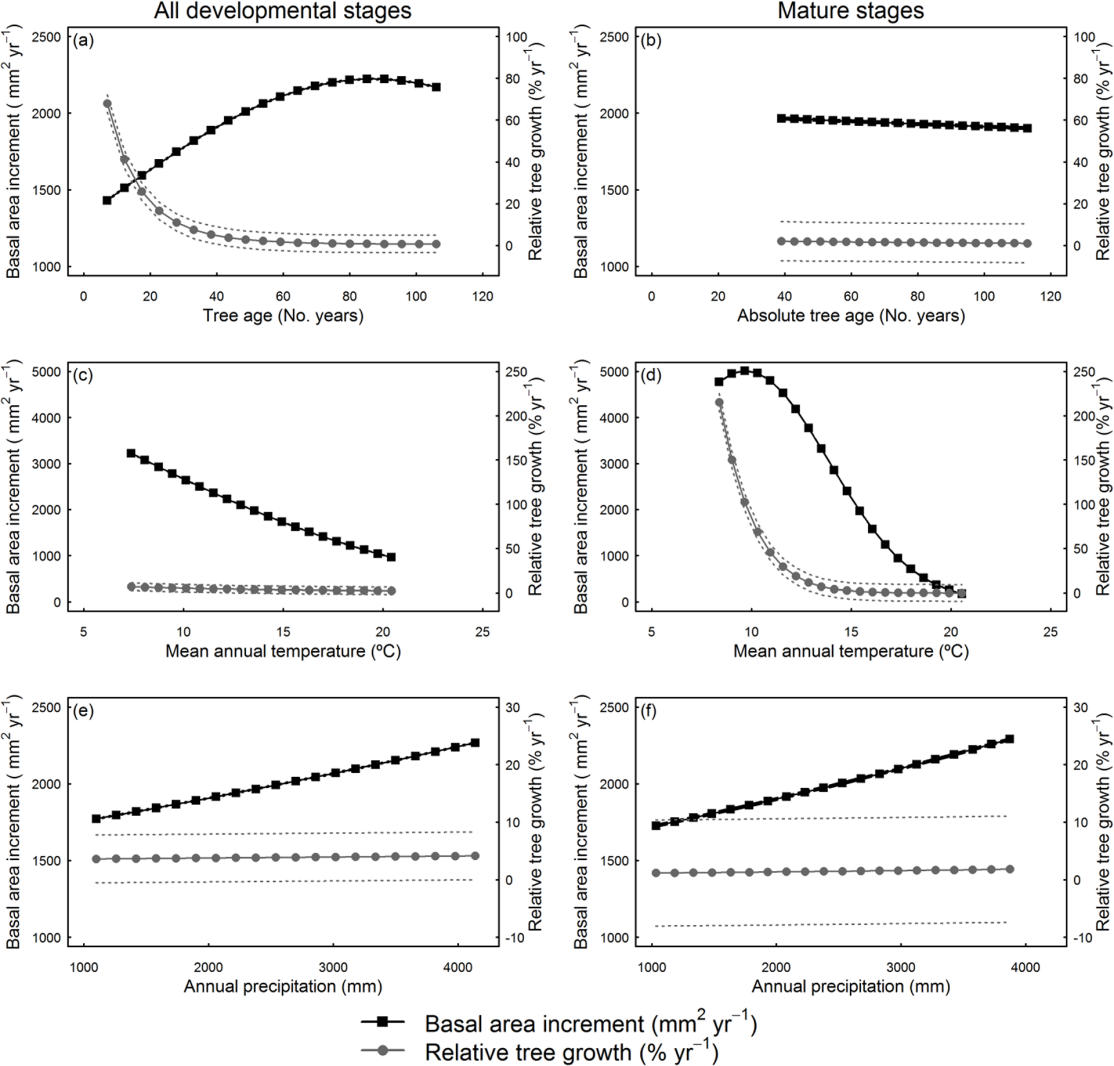
Predicted basal area increment and relative tree growth along against age, temperature and precipitation. Predicted tree basal area increment (m^2^ yr^-1^) and relative tree growth (% yr^-1^) for all data and mature stage data in relation to: ((a) and (b), respectively) tree age (No. years), ((c) and (d), respectively) mean annual temperature (°C), and ((e) and (f), respectively) annual precipitation (mm).

Tree growth responses with age varied the sign and magnitude depending on stand development (see Fig 4 and parameter values in Table 3). In models parameterized using all developmental stages we observed that basal area increment increases along the entire tree age gradient (Fig 4A). Relative tree growth was greatest for young trees (*TA* < 20 years), levelling out at larger tree ages (Fig 4A). However, in models parameterised using mature stage data, there was almost no effect of tree age on tree growth (Fig 4B).

**Fig 5:**
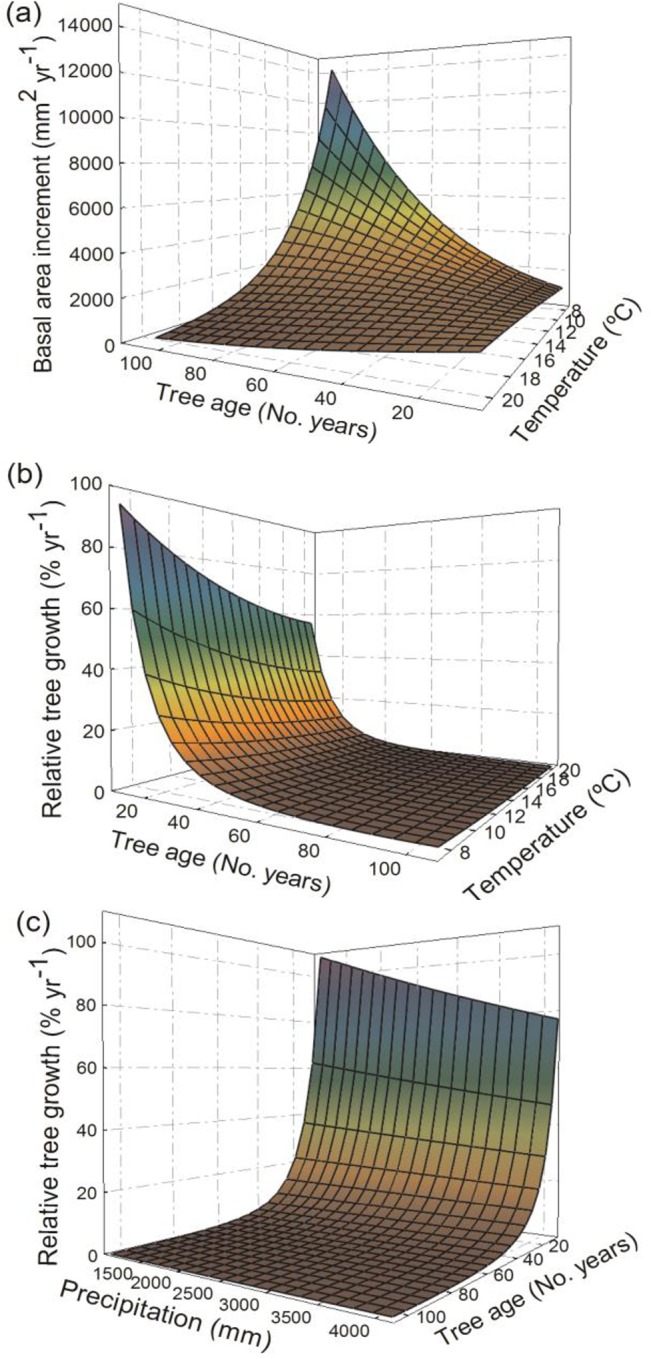
Interactive effects of tree age and climate on basal area increment and relative tree growth. Predicted (a) basal area increment (m^2^ yr^-1^) and (b) relative tree growth (% yr^-1^) along tree age (No. years) and mean annual temperature (°C); and (c) relative tree growth along tree age (No. years) and mean annual precipitation (mm) in models parameterised using data from all developmental stages.

At high mean annual temperatures both absolute and relative tree growth were lowest, independently of the data considered (i.e. both all developmental stages together and mature stage alone, see Fig **4**C and 4D). Furthermore, the interactions between tree age and mean annual temperature indicated that at high mean annual temperature both absolute and relative tree growth responses are suppressed along the entire tree age gradient (see Fig **5**A and 5B). The reduction of absolute tree growth caused by increasing mean annual temperature was much higher in old trees (i.e. reductions in absolute tree growth along increased temperature were greater for old than young trees, Fig 5A), but in relative tree growth variation along temperature was greater in young trees (i.e. reductions in relative tree growth with increased temperature were greater for young than old trees, Fig 5B).

Regarding annual precipitation effects on tree growth, although its effect was lower than the one observed for mean annual temperature, we observed a positive linear relationship with annual precipitation for absolute tree growth in all forest types (see Fig **4**E and 4F). However, for relative tree growth there was no variation in growth responses with annual precipitation, except for small trees in models parameterized with all developmental stages, where higher growth responses were observed at low annual precipitation levels (Fig 5C).

## Discussion

Tree age was the most important factor determining absolute and relative tree growth, but this relationship was not significant in mature stand development stages, confirming that stand development and stand structure play a crucial role driving the sign and magnitude of age-related responses (e.g. [8, 50]). Mean annual temperature was more important than annual precipitation shaping both absolute and relative tree growth. Increased temperature caused a sharp decrease in tree growth and age-related relationships were neutral at high mean annual temperature levels. Furthermore, under increased temperature, young trees were the most sensitive in relative growth terms, but old trees were the most sensitive in absolute growth terms. These patterns suggest that a warming climate is likely to cause a strong reduction of growth in subtropical *Pinus taiwanensis* stands at high temperatures (e.g. lowland forests), potentially altering future carbon storage due to reduced absolute growth of large trees and constraining relative growth of young trees.

### Effects of Stand Development on Age-Related Growth Responses

Our results demonstrate that absolute tree growth increases and relative tree growth decreases with tree age up to 80 years during stand development (see Fig 4A). The positive effect of tree age on absolute growth agrees with recent evidence found worldwide [7]. Positive tree growth with stand age and/or size have been related to tree physiological adjustments as stand develops as more efficient leaf organization and increased leaf packing within the crown (i.e. old trees tend to maximize the light captured, [20]). Therefore, increases in total leaf area may compensate reductions in photosynthetic or growth efficiency, suggesting that carbon limitation is not leading to age-related growth decline [18], although the negative effect of nutrient and water supply on tree growth is more controversial (see [15, 51]).

Despite the increased absolute growth with tree size observed as stands develop; the relationship became not significant and slightly negative at mature stages (see Fig 4B and parameters in Table 3). During stand development there are changes in stand structure (e.g. vegetation height, tree density, evenness) that determine nutrient and light availability [52, 53]. Therefore, the sign and magnitude of age-related growth responses at the individual level may change during stand development depending on the competitive environment [8, 22]. However, the slight decline of growth found with tree age is consistent with recent evidence that suggests more neutral relationships with tree age due to physiological and structural adjustments at the tree level [20, 50] that may be compensated by a greater likelihood of cavitation in the xylem of large trees (i.e. hydraulic failure, see [15]). Our results bring further evidence to unify the controversial patterns of growth at the tree level, because models parameterized at mature stages may have relatively similar stand structure conditions (e.g. medium to high stand density and heterogeneity) where productivity declines with age have been largely observed [cf. 7, 13].

### Effects of Climate on Tree Growth Responses

We found that rising temperature had a negative effect on absolute and relative tree growth, much larger than the effect of precipitation. This result indicates that high temperatures are a climate constraint to *Pinus taiwanensis* growth, and the intensity of growth reduction may be exacerbated in the warmest areas of its range (at its lower altitudinal distribution limit, see Fig 1). Other authors have already found that absolute tree growth rates are negatively correlated with increased temperature in tropical forests [54–56]. Furthermore, the negative effect of rising temperature in tropical forests seems particularly strong for evergreen species [29]. Under high mean annual temperatures, leaf net photosynthesis can be highly altered due to increased plant respiration, stomatal sensitivity to increased vapour deficit, and changes in biochemical processes [57]. There is an intense debate regarding whether increased carbon fertilization can offset reduced productivity due to increased temperatures in tropical forests [58]. However, there is increasing evidence that higher temperatures can exceed the temperature threshold for photosynthesis and cause reductions in CO_2_ assimilation and growth in tropical and subtropical forests (e.g. [59]).

The largest growth declines with mean annual temperature occurred in mature stage forests (see Fig **4**C and 4D), suggesting that areas where mean annual temperature is the highest (e.g. lowland forests) and canopies are particularly dense could suffer particularly reduced growth. Other authors have already observed that growth responses with temperature are also dependent on stand structural conditions [50], because high competition is a key driver determining tree growth patterns (e.g. [24]). *Pinus taiwanensis* is able to colonise even under extreme climatic conditions and plays a crucial role in stabilising slopes after landslides in this typhoon prone region [39]. However, if climate continues to warm (a net mean temperature increment of *c*. 1°C from 1960 to 2010 is observed in Fig 2C), our results indicate that it may cause strongly reduced tree growth of *P*. *taiwanensis* forests.

Annual precipitation had a positive effect on absolute tree growth, suggesting that increments in water availability can lead to growth pulses (see Figs 1 and 3). The lower importance of annual precipitation than temperature determining tree growth agrees with previous suggestions regarding the relatively low correlation between productivity and rainfall in tropical forests [60]. However, although we found that annual precipitation had a relatively low importance, temporal changes in rainfall patterns can result in absolute tree growth increments (see Fig 2B and Fig **4**E and 4F). Relative tree growth was higher at low values of annual precipitation in young trees, which can be due to the fact that high precipitation levels can cause anaerobic soil conditions or increase nutrient limitation in tropical forests, and thus, reduce growth [61].

### Interactive Effects between Climate and Age on Growth

We observed strong interactions between mean annual temperature and tree age, which suggest that reductions of absolute tree growth under increased temperature disproportionally affect old trees (Fig 5A) whereas young trees were more sensitive in terms of relative tree growth (Fig 5B). On the one hand, our results suggest that large and old trees are able to store large amounts of biomass [7], but old trees may have particularly reduced growth under climate warming [9] as observed in the steep drop in absolute tree growth with increased mean annual temperature. This result agrees with previous studies, which found a higher sensitivity of absolute tree growth to climate in old trees and hypothesized that there is an increased probability of hydraulic failure in large individuals (e.g. [31, 62]). On the other hand, the larger sensitivity of relative tree growth to increased temperature in young trees agrees point out that these early stages can be particularly impacted by rising temperatures. Other authors have also found a higher sensitivity of young trees for relative tree growth, and this result has been related to a more conservative use of water (e.g. [8, 10]). Overall, our results suggest that growth in old trees may be more resilient to climate warming than for young trees, but small changes in growth can cause steep drops in absolute tree growth under increased temperature.

## Conclusions

In this study we provide further evidence to unify contrasting results regarding both the sign and the magnitude of growth responses with tree age. We suggest that the different results obtained at the tree level may be due to differential growth responses depending on forest development stages and therefore they may change depending on the competitive environment, stand heterogeneity, nutrient availability and species composition [8, 50].

Overall, the strong negative effect of climate warming on individual tree growth suggests that these forests are highly vulnerable to increased temperature. Rising temperatures due to climate change have been already identified as critical for determining altitudinal species range shifts in tropical forests [30, 63]. Furthermore, we found that growth reductions have the potential to disproportionately affect warm areas and mature stands. Therefore, it is critical to understand the impact of potential growth reductions throughout the range of *P*. *taiwanensis* since it may negatively impact on the essential ecosystem services that this species provides [39]. Any growth reduction occurring as a consequence of climate warming is also likely to lead to altitudinal changes of species distributions and competitive ability, thereby impacting community structure and diversity below the treeline, as has been witnessed for plant species at higher altitudes in this region [64].

Our results agree with recent suggestions of the importance of large trees for absolute tree growth and, therefore, on the carbon cycle [7, 10]. Absolute growth increments may be reduced under increased temperature, which might compromise the role of forests as a future carbon sink under climate change scenarios. Furthermore, young trees were highly sensitive in relative growth terms to temperature increases, suggesting that early establishment stages may constitute a bottleneck for persistence as the climate warms.

## Supporting Information

S1 Fig Box-whisker plots of stand basal area increment and relative tree growth with predictor variables in models developed using all data. ((a) and (b), respectively) tree age (No. years), ((c) and (d), respectively) mean annual temperature (°C) and ((e) and (f), respectively) annual precipitation (mm).
(DOCX)

S2 Fig Box-whisker plots of stand basal area increment and relative tree growth with predictor variables in mature stages. ((a) and (b), respectively) tree age (No. years), ((c) and (d), respectively) mean annual temperature (°C) and ((e) and (f), respectively) annual precipitation (mm).
(DOCX)

S3 Fig Residuals of basal area increment models. Scatterplot of residual versus predicted log of basal area increment and histogram of the residuals residual for the final models using all data ((a) and (b), respectively) and using data corresponding to mature stages ((c) and (d), respectively).
(DOCX)

S4 Fig Residuals of relative tree growth models. Scatterplot of residual versus predicted log of relative tree growth (% yr^-1^) and histogram of the residuals residual for the final models using all data ((a) and (b), respectively) and using data corresponding to mature stages ((c) and (d), respectively).
(DOCX)

S1 Table Selected studies of age- and size- dependent growth responses reported at the individual tree level. (DOCX)

S2 Table Plant community composition summary in each study site. Functional group (FG) composition is summarised as tree, shrub, herb or fern. Woody species richness refers to trees and shrubs in each site.
(DOCX)

S3 Table Summary statistics of ring width index chronologies of *Pinus taiwanensis* for the common interval 1975–2009. (DOCX)

S4 Table Comparison of basal area increment and relative tree growth models parameterized using Akaike Information Criterion (AIC). The models were parameterized using one predictor variable (i.e. tree age, mean annual temperature or annual precipitation) and different functional forms (i.e. linear or quadratic).
(DOCX)

S5 Table Comparison of alternative basal area increment models and relative tree growth models. We tested seven candidate models without interactions and three models that include all possible pair-wise interactions between tree age and climatic variables using Akaike Information Criterion (AIC).
(DOCX)
