# Supplementary material for: Climatic Stress during Stand Development Alters the Sign and Magnitude of Age-Related Growth Responses in a Subtropical Mountain Pine
Source: PLoS One. 2015 May 14;10(5):e0126581. doi: 10.1371/journal.pone.0126581 (PMC4431836; doi:10.1371/journal.pone.0126581)
Supplement: S2 Fig — ((a) and (b), respectively) tree age (No. years), ((c) and (d), respectively) mean annual temperature (°C) and ((e) and (f), respectively) annual precipitation (mm). (DOCX) [file pone.0126581.s002.docx]

**
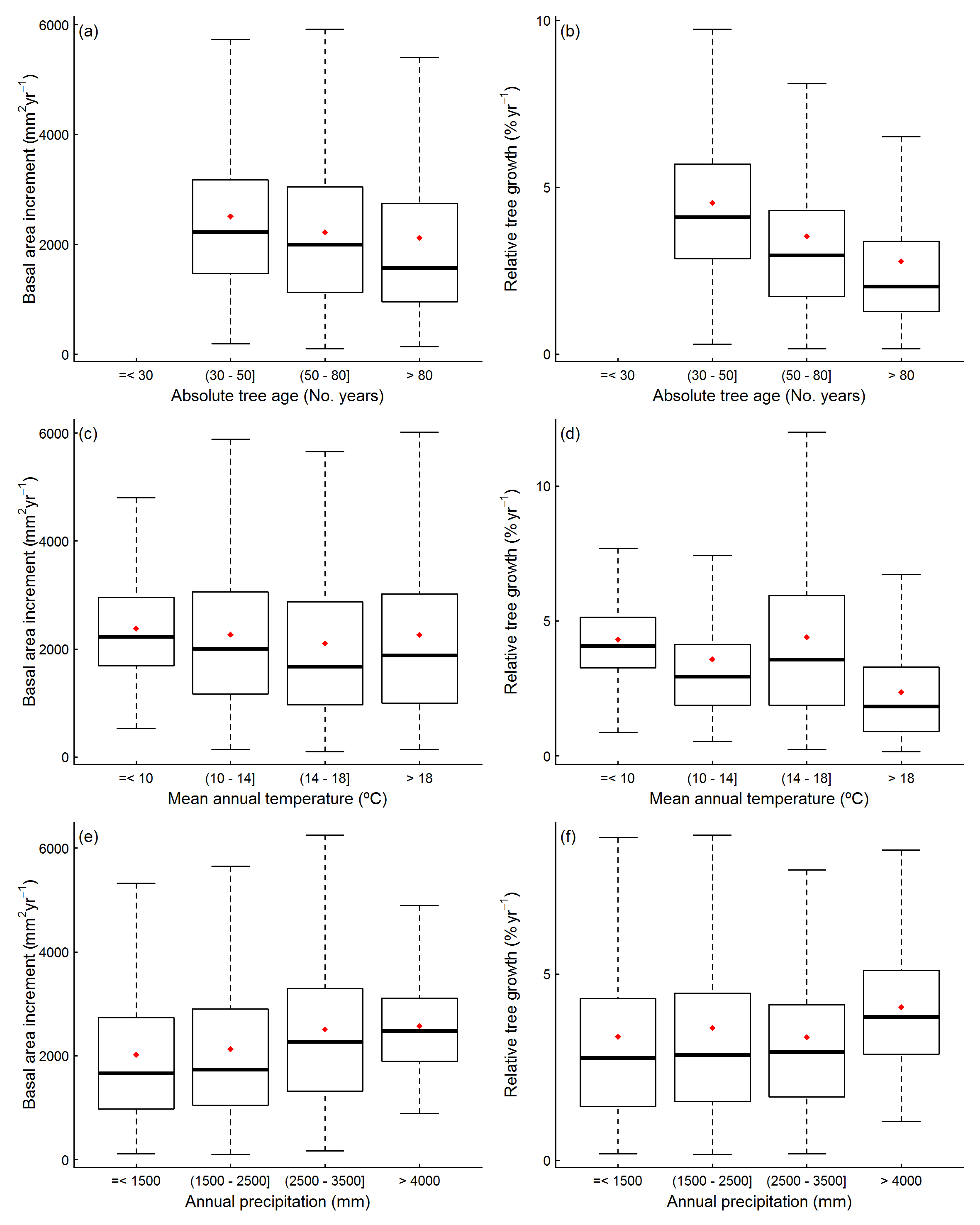
**

**S2 Figure. Box-whisker plots of stand basal area increment and relative tree growth with predictor variables in mature stages.** (**(a)** and **(b)**, respectively) tree age (No. years), (**(c)** and **(d)**, respectively) mean annual temperature (ºC) and (**(e)** and **(f)**, respectively) annual precipitation (mm).
