# Supplementary material for: Climatic Stress during Stand Development Alters the Sign and Magnitude of Age-Related Growth Responses in a Subtropical Mountain Pine
Source: PLoS One. 2015 May 14;10(5):e0126581. doi: 10.1371/journal.pone.0126581 (PMC4431836; doi:10.1371/journal.pone.0126581)
Supplement: S3 Fig — Scatterplot of residual versus predicted log of basal area increment and histogram of the residuals residual for the final models using all data ((a) and (b), respectively) and using data corresponding to mature stages ((c) and (d), respectively). (DOCX) [file pone.0126581.s003.docx]

**
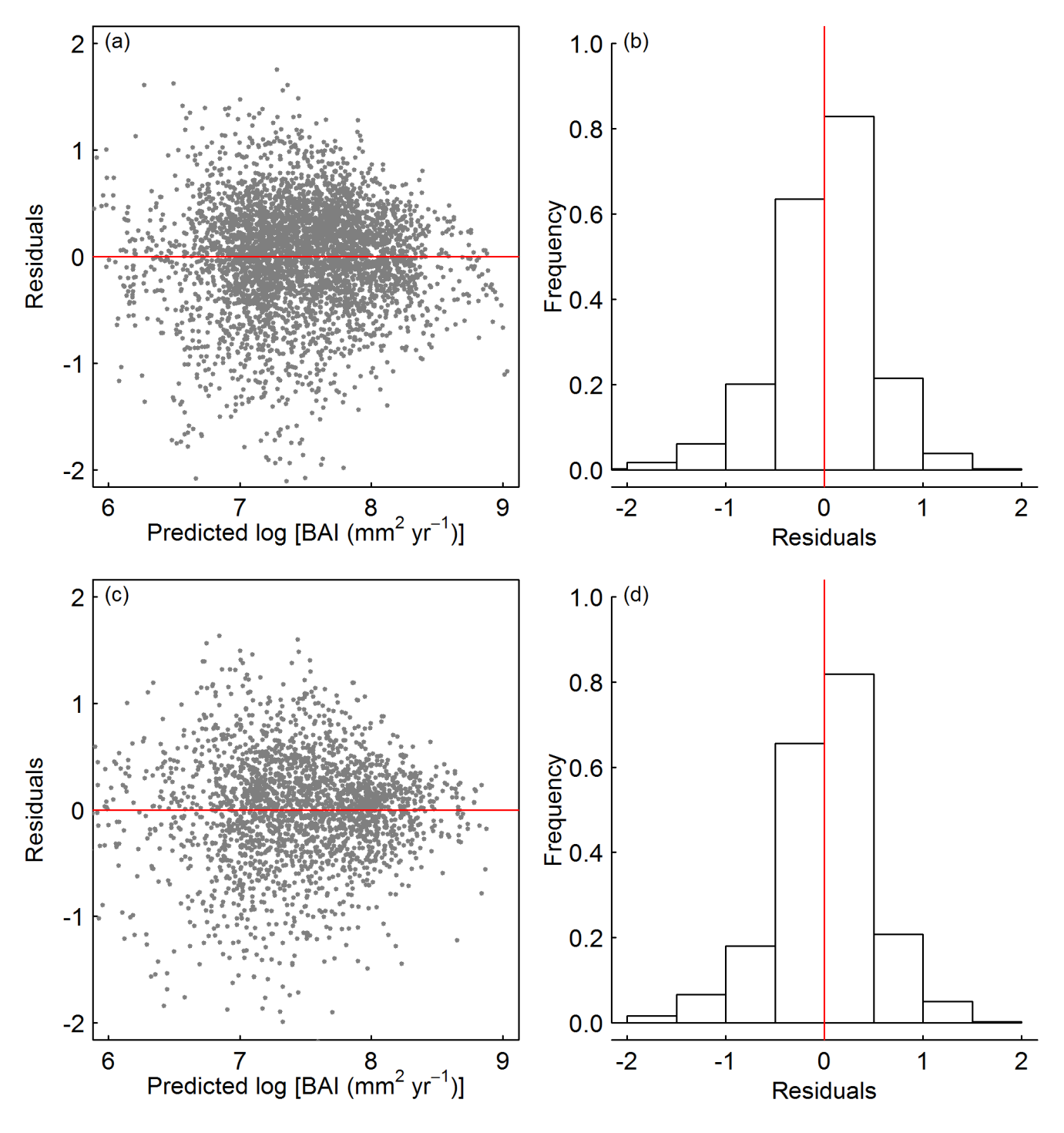
**

**S3 Figure. Residuals of basal area increment models.** Scatterplot of residual versus predicted log of basal area increment and histogram of the residuals residual for the final models using all data (**(a)** and **(b)**, respectively) and using data corresponding to mature stages (**(c)** and **(d)**, respectively).
