# Supplementary material for: Climatic Stress during Stand Development Alters the Sign and Magnitude of Age-Related Growth Responses in a Subtropical Mountain Pine
Source: PLoS One. 2015 May 14;10(5):e0126581. doi: 10.1371/journal.pone.0126581 (PMC4431836; doi:10.1371/journal.pone.0126581)
Supplement: S4 Fig — Scatterplot of residual versus predicted log of relative tree growth (% yr-1) and histogram of the residuals residual for the final models using all data ((a) and (b), respectively) and using data corresponding to mature stages ((c) and (d), respectively). (DOCX) [file pone.0126581.s004.docx]

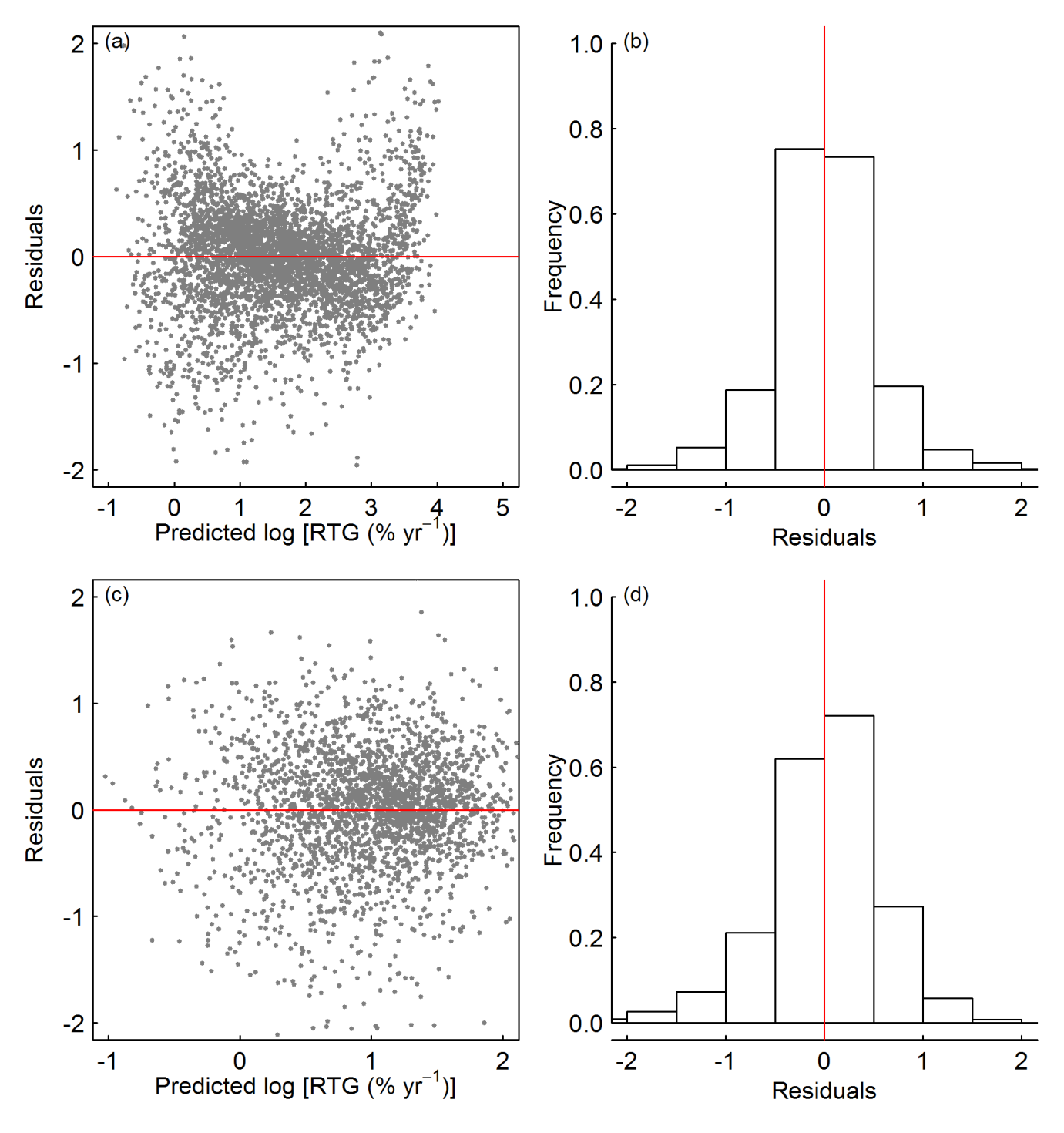


**S4 Figure. Residuals of relative tree growth models.** Scatterplot of residual versus predicted log of relative tree growth (% yr^-1^) and histogram of the residuals residual for the final models using all data (**(a)** and **(b)**, respectively) and using data corresponding to mature stages (**(c)** and **(d)**, respectively).
