# Supplementary material for: Climatic Stress during Stand Development Alters the Sign and Magnitude of Age-Related Growth Responses in a Subtropical Mountain Pine
Source: PLoS One. 2015 May 14;10(5):e0126581. doi: 10.1371/journal.pone.0126581 (PMC4431836; doi:10.1371/journal.pone.0126581)
Supplement: S1 Table — (DOCX) [file pone.0126581.s005.docx]

**S1 Table. Selected studies of age- and size- dependent growth responses reported at the individual tree level.**

| **Reference*** | **Level** | **Response variable** | **Explanatory variable** | **Relationship observed** | **Type of data/analysis** | **Type of species/forest** | **Key findings/Causes suggested** |
| --- | --- | --- | --- | --- | --- | --- | --- |
| Coomes *et al.* (2014) | Tree (both) | Annual stem diameter growth | Tree size (canopy area index, temperature, precipitation) | Hump-shaped or reached an asymptote | Permanently marked plots (Hierarchical Bayesian models) | New Zealand’s natural forests (high-diverse) | Biomass growth continues to increase with size, but diameter growth often peaks at immediate tree sizes |
|  | Stand (both) | Aboveground wood production | Mean size (composition, packing, diversity, temperature, precipitation) | Increase, slight decline after accounting basal area | Permanently marked plots (Hierarchical Bayesian models) | New Zealand’s natural forests (high-diverse) | Increases due to colonisation. Individual physiological, structural and anatomical adjustments may prevent declines in growth with size |
| Prior & Bowman (2014) | Tree | Stem diameter growth | Tree size (temperature) | Hump-shaped | Linear mixed effects models | Eucalypt (temperate Australia) | Hump-shaped increase where large trees are more sensitive to temperature increases |
| Stephenson *et al.* (2014) | Tree | Mass growth rate | Tree size | Increase | National forest inventories (Bayesian models) | 403 tropical and temperate species | Large effect of old trees on carbon cycle |
| Coomes *et al.* (2012) | Tree (both) | Biomass production | Tree size (canopy area index, altitude, soil) | Increase | Permanently marked plots (linear mixed-effects models) | Beech forest (New Zealand) | Individual trees can increase productivity by adjusting the positioning, nutrient content and angle of leaves within canopies. |
|  | Stand (both) | Biomass production | Mean biomass (canopy area index, altitude, soil) | Decline | Patch dynamic model | Beech forest (New Zealand) | Disturbance have a key role determining carbon cycle, because of losses of biomass due to dead trees |
| Sillet *et al.* (2010) | Tree | Ground measures of annual absolute growth, and wood production of the trunk and whole crown | Age, size | Decline or no change, but wood production increase | Ordinary least square regression | 43 un-suppressed individuals of E*ucalyptus regnans* and *Sequoia sempervirens* | Wood production was highly explained by tree size and increased production in appendances |
| Phillips, Buckley & Tissue (2008) | Tree | Ring width | Year | Monotonic increase | Trees chronologies (tree level) and process-based model (stand level) | *Pseudotsuga menziesii* (Temperate old growth forest ) | Growth increased in old growth forests |
| Xu *et al.* (2012) | Tree (both) | Above-ground biomass | Tree age | No evidence | Tree ring growth | *Quercus*-dominated deciduous forest | Not age-related physiological constraints or reductions in resource use efficiency |
|  | Stand (both) | Above-ground biomass | Tree age | Decline | Long-term plots | *Quercus*-dominated deciduous forest | Death of large and dominant trees was the cause of decline |
| Piper & Fajardo (2011) | Tree | Basal area increment (Carbon gain and demand balance) | Tree age (drought constrain) | Hump-shaped | Observational (ANOVA) | Deciduous temperate species *Nothofagus pumilio* (monospecific forests) | Carbon limitation was not the cause age-related decline |
| Gomez-Aparicio *et al.* (2011) | Tree | Diameter increase | Tree size (competition, climate) | Hump-shaped | Spanish forest inventory (Maximum likelihood models) | 15 Mediterranean and temperate species | Size-growth relationship was species dependent. The magnitude of the effect was larger for conifers than for broadleaved species. |
| Caspersen *et al.* (2001) | Tree (both) | Tree growth | Tree diameter (competition) | Hump-shaped | Canadian forest inventory (cohort-based canopy competition model, ‘‘CAIN’’) | Temperate, boreal | Age-dependent responses varies with species composition and evenness |
|  | Stand (both) | Net productivity | Size distribution (stand effect, competition) | Hump-shaped | Simulated model | Temperate, boreal | Both growth and mortality exert an influence on stand productivity |

**References**

Caspersen JP, Vanderwel MC, Cole WG, Purves DW. How stand productivity results from size- and competition-dependent growth and mortality. PloS ONE. 2011;6(12):e28660.

Coomes D, Flores O, Holdaway R, Jucker T, Lines ER, Vanderwel MC. Wood production response to climate change will depend critically on forest composition and structure. Global Change Biol. 2014;20(12):3632-45.

Coomes DA, Holdaway RJ, Kobe RK, Lines ER, Allen RB. A general integrative framework for modelling woody biomass production and carbon sequestration rates in forests. J Ecol. 2012;100(1):42-64.

Gómez-Aparicio L, García-Valdés R, Ruiz-Benito P, Zavala MA. Disentangling the relative importance of climate, size and competition on tree growth in Iberian forests: implications for management under global change. Global Change Biol. 2011;17(7):2400-14.

Phillips NG, Buckley TN, Tissue DT. Capacity of old trees to respond to environmental change. J Integr Plant Biol. 2008;50(11):1355-64.

Piper FI, Fajardo A. No evidence of carbon limitation with tree age and height in Nothofagus pumilio under Mediterranean and temperate climate conditions. Ann Bot. 2011;108(5):907-17.

Prior LD, Bowman DMJS. Big eucalypts grow more slowly in a warm climate: evidence of an interaction between tree size and temperature. Global Change Biol. 2014;20(9):2793–9.

Sillett SC, Van Pelt R, Koch GW, Ambrose AR, Carroll AL, Antoine ME, et al. Increasing wood production through old age in tall trees. For Ecol Manage. 2010;259(5):976-94.

Stephenson NL, Das AJ, Condit R, Russo SE, Baker PJ, Beckman NG, et al. Rate of tree carbon accumulation increases continuously with tree size. Nature. 2014;507:90-3.

Xu C, Turnbull MH, Tissue DT, Lewis JD, Carson R, Schuster WSF, et al. Age-related decline of stand biomass accumulation is primarily due to mortality and not to reduction in NPP associated with individual tree physiology, tree growth or stand structure in a Quercus-dominated forest. J Ecol. 2012;100(2):428-40.
