# Supplementary material for: Climatic Stress during Stand Development Alters the Sign and Magnitude of Age-Related Growth Responses in a Subtropical Mountain Pine
Source: PLoS One. 2015 May 14;10(5):e0126581. doi: 10.1371/journal.pone.0126581 (PMC4431836; doi:10.1371/journal.pone.0126581)
Supplement: S2 Table — Functional group (FG) composition is summarised as tree, shrub, herb or fern. Woody species richness refers to trees and shrubs in each site. (DOCX) [file pone.0126581.s006.docx]

**S2 Table. Plant community composition summary in each study site.** Functional group (FG) composition is summarised as tree, shrub, herb or fern. Woody species richness refers to trees and shrubs in each site.

| **Bottom (695 m a.s.l.)**  **Species richness = 36 (Woody species richness = 27)** | | | |  |
| --- | --- | --- | --- | --- |
| **Species** | | **Family** | | **FG** |
| *Ilex asprella* (Hook. & Arn.) Champ. | | *Aquifoliaceae* | | Tree |
| *Daphniphyllum glaucescens* Blume subsp. *oldhamii* (Hemsl.) | | *Daphniphyllaceae* | | Tree |
| *Diospyros morrisiana* Hance | | *Ebenaceae* | | Tree |
| *Elaeocarpus sylvestris* (Lour.) Poir. | | *Elaeocarpaceae* | | Tree |
| *Mallotus japonicus* (Thunb.) Müll. Arg. | | *Euphoribiaceae* | | Tree |
| *Castanopsis kawakamii* Hayata | | *Fagaceae* | | Tree |
| *Cyclobalanopsis glauca* (Thunb.) Oerst. | | *Fagaceae* | | Tree |
| *Liquidambar formosana* Hance | | *Hamamelidaceae* | | Tree |
| *Cinnamomum insulari-montanum* Hayata | | *Lauraceae* | | Tree |
| *Litsea acuminata* (Blume) Kurata | | *Lauraceae* | | Tree |
| *Litsea hypophaea* Hayata | | *Lauraceae* | | Tree |
| *Neolitsea aciculata* (Blume) Koidz. var. *variabillima* (Hayata) J. C. Liao | | *Lauraceae* | | Tree |
| *Melicope semecarpifolia* (Merr.) T. Hartley | | *Rutaceae* | | Tree |
| *Ecdysanthera rosea* Hook. & Arn. | | *Apocynaceae* | | Shrub |
| *Sarcandra glabra* (Thunb.) Nakai | | *Chloranthaceae* | | Shrub |
| *Rhododendron oldhamii* Maxim. | | *Ericaceae* | | Shrub |
| *Glochidion rubrum* Blume | | *Euphoribiaceae* | | Shrub |
| *Lindera communis* Hemsl. | | *Lauraceae* | | Shrub |
| *Bauhinia championii* (Benth.) Benth. | | *Leguminosae* | | Shrub |
| *Derris laxiflora* Benth. | | *Leguminosae* | | Shrub |
| *Maesa perlaria* (Lour.) Merr. var. *formosana* (Mez) Yuen P. Yang | | *Myrsinaceae* | | Shrub |
| *Syzygium formosanum* (Hayata) Mori | | *Myrtaceae* | | Shrub |
| *Rubus corchorifolius* L. f. | | *Rosaceae* | | Shrub |
| *Gardenia jasminoides* Ellis | | *Rubiaceae* | | Shrub |
| *Mussaenda pubescens* W. T. Aiton | | *Rubiaceae* | | Shrub |
| *Psychotria rubra* (Lour.) Poir. | | *Rubiaceae* | | Shrub |
| *Clerodendrum cyrtophyllum* Turcz. | | *Verbenaceae* | | Shrub |
| *Amischotolype hispida* (Less. & A. Rich.) D. Y. Hong | | *Commelinaceae* | | Herb |
| *Arundinaria usawai* Hayata | | *Gramineae (Poaceae)* | | Herb |
| *Ophiorrhiza japonica* Blume | | *Rubiaceae* | | Herb |
| *Tectaria phaeocaulis* (Rosenst.) C. Chr. | | *Aspidiaceae* | | Fern |
| *Dryopteris formosana* (H. Christ) C. Chr. | | *Dryopteridaceae* | | Fern |
| *Dicranopteris linearis* (Burm. f.) Underw. | | *Gleicheniaceae* | | Fern |
| *Nephrolepis auriculata* (L.) Trimen | | *Oleandraceae* | | Fern |
| *Pteris semipinnata* L. | | *Pteridaceae* | | Fern |
| *Lygodium japonicum* (Thunb.) Sw. | | *Schizaeaceae* | | Fern |
| **Low (1,213 m a.s.l.)**  **Species richness = 29 (Woody species richness = 20)** | | | | |
| **Species** | **Family** | | **FG** | |
| *Calocedrus macrolepis* Kurz var. *formosana* (Florin) W. C. Cheng & L. K. Fu | *Cuprassaceae* | | Tree | |
| *Cyclobalanopsis glauca* (Thunb.) Oerst. | *Fagaceae* | | Tree | |
| *Quercus variabilis* Blume | *Fagaceae* | | Tree | |
| *Liquidambar formosana* Hance | *Hamamelidaceae* | | Tree | |
| *Cinnamomum camphora* (L.) J. Presl | *Lauraceae* | | Tree | |
| *Fraxinus griffithii* C. B. Clarke | *Oleaceae* | | Tree | |
| *Prunus campanulata* Maxim. | *Rosaceae* | | Tree | |
| *Cryptomeria japonica* (Thunb. ex L. f.) D. Don | *Taxodiaceae* | | Tree | |
| *Cunninghamia lanceolata* (Lamb.) Hook. | *Taxodiaceae* | | Tree | |
| *Zelkova serrata* (Thunb.) Makino | *Ulmaceae* | | Tree | |
| *Glochidion rubrum* Blume | *Euphoribiaceae* | | Shrub | |
| *Akebia longeracemosa* Matsum. | *Lardizabalaceae* | | Shrub | |
| *Pueraria lobata* (Willd.) Ohwi subsp. *thomsonii* (Benth.) H. Ohashi & Tateishi | *Leguminosae* | | Shrub | |
| *Ardisia cornudentata* Mez subsp. *morrisonensis* (Hayata) Yuen P. Yang | *Myrsinaceae* | | Shrub | |
| *Maesa perlaria* (Lour.) Merr. var. *formosana* (Mez) Yuen P. Yang | *Myrsinaceae* | | Shrub | |
| *Clematis grata* Wall. | *Ranunculaceae* | | Shrub | |
| *Tetradium glabrifolium* (Champ. ex Benth.) T. Hartley | *Rutaceae* | | Shrub | |
| *Callicarpa formosana* Rolfe | *Verbenaceae* | | Shrub | |
| *Ampelopsis brevipedunculata* (Maxim.) Trautv. var. *hancei* (Planch.) Rehder | *Vitaceae* | | Shrub | |
| *Tetrastigma formosanum* (Hemsl.) Gagnep. | *Vitaceae* | | Shrub | |
| *Trichodesma calycosum* Collett & Hemsl. | *Boraginaceae* | | Herb | |
| *Humata griffithiana* (Hook.) C. Chr. | *Davalliaceae* | | Herb | |
| *Miscanthus sinensis* Anderss var. *transmorrisonensis*（Hayata）Y. Lee. | *Gramineae (Poaceae)* | | Herb | |
| *Belamcanda chinensis* (L.) DC. | *Iridaceae* | | Herb | |
| *Asparagus cochinchinensis* (Lour.) Merr. | *Liliaceae* | | Herb | |
| *Dryopteris formosana* (H. Christ) C. Chr. | *Dryopteridaceae* | | Fern | |
| *Nephrolepis auriculata* (L.) Trimen | *Oleandraceae* | | Fern | |
| *Pyrrosia lingua* (Thunb.) Farw. | *Polygonaceae* | | Fern | |
| *Onychium contiguum* C. Hope | *Pteridaceae* | | Fern | |
| **Medium (2,166 m a.s.l.)**  **Species richness = 23 (Woody species richness = 18)** | | | | |
| **Species** | **Family** | | **FG** | |
| *Acer kawakamii* Koidz. | *Aceraceae* | | Tree | |
| *Acer morrisonense* Hayata | *Aceraceae* | | Tree | |
| *Elaeocarpus japonicus* Siebold & Zucc. | *Elaeocarpaceae* | | Tree | |
| *Rhododendron formosanum* Hemsl. | *Ericaceae* | | Tree | |
| *Pasania hancei* (Benth.) Schottky var. *ternaticupula* (Hayata) J. C. Liao | *Fagaceae* | | Tree | |
| *Neolitsea acuminatissima* (Hayata) Kaneh. & Sasaki | *Lauraceae* | | Tree | |
| *Podocarpus fasciculus* de Laub. | *Podocarpaceae* | | Tree | |
| *Meliosma squamulata* Hance | *Sabiaceae* | | Tree | |
| *Camellia brevistyla* (Hayata) Cohen-Stuart | *Theaceae* | | Tree | |
| *Gordonia axillaris* (Roxb.) Dietr. | *Theaceae* | | Tree | |
| *Ternstroemia gymnanthera* (Wight & Arn.) Sprague | *Theaceae* | | Tree | |
| *Dendropanax dentiger* (Harms ex Diels) Merr. | *Araliaceae* | | Shrub | |
| *Pieris taiwanensis* Hayata | *Ericaceae* | | Shrub | |
| *Rhododendron oldhamii* Maxim. | *Ericaceae* | | Shrub | |
| *Pachycentria formosana* Hayata | *Melastomataceae* | | Shrub | |
| *Ardisia cornudentata* Mez subsp. *morrisonensis* (Hayata) Yuen P. Yang | *Myrsinaceae* | | Shrub | |
| *Myrsine stolonifera* (Koidz.) Walker | *Myrsinaceae* | | Shrub | |
| *Smilax china* L. | *Smilacaceae* | | Shrub | |
| *Ascocentrum pumilum* (Hayata) Schltr. | *Orchidaceae* | | Herb | |
| *Lepisorus thunbergianus* (Kaulf.) Ching | *Polygonaceae* | | Herb | |
| *Davallia mariesii* T. Moore *ex* Baker | *Davalliaceae* | | Fern | |
| *Diplopterygium glaucum* (Houtt.) Nakai | *Gleicheniaceae* | | Fern | |
| *Pyrrosia lingua* (Thunb.) Farw. | *Polypodiaceae* | | Fern | |
| **High (2,444 m a.s.l.) Species richness = 11 (Woody species richness = 7)** | | | | |
| **Species** | **Family** | | **FG** | |
| *Acer kawakamii* Koidz. | *Aceraceae* | | Tree | |
| *Pinus morrisonicola* Hayata | *Pinaceae* | | Tree | |
| *Tsuga chinensis* (Franch.) Pritz. ex Diels var. formosana (Hayata) H. L. Li & H. Keng | *Pinaceae* | | Tree | |
| *Prunus campanulata* Maxim. | *Rosaceae* | | Tree | |
| *Sambucus chinensis* Lindl. | *Caprifoliaceae* | | Shrub | |
| *Photinia niitakayamensis* Hayata | *Rosaceae* | | Shrub | |
| *Eurya crenatifolia* (Yamam.) Kobuski | *Theaceae* | | Shrub | |
| *Senecio scandens* Buch.-Ham. ex D. Don. | *Compositae* | | Herb | |
| *Miscanthus sinensis* Anderss var. t*ransmorrisonensis*（Hayata）Y. Lee. | *Gramineae (Poaceae)* | | Herb | |
| *Polygonum chinense* L. | *Polygonaceae* | | Herb | |
| *Urtica thunbergiana* Siebold & Zucc. | *Urticaceae* | | Herb | |
| **Top (2,941 m a.s.l.)**  **Species richness = 5 (Woody species richness = 3)** | | | | |
| **Species** | **Family** | | **FG** | |
| *Berberis kawakamii* Hayata | *Berberidaceae* | | Shrub | |
| *Rhododendron rubropilosum* Hayata var. *taiwanalpinum* (Ohwi) S. Y. Lu, Yuen P. Yang & Y. H. Tseng | *Ericaceae* | | Shrub | |
| *Eurya glaberrima* Hayata | *Theaceae* | | Shrub | |
| *Miscanthus sinensis* Anderss var. *transmorrisonensis*（Hayata）Y. Lee. | *Gramineae* | | Herb | |
| *Yushania niitakayamensis* (Hayata) Keng f. | *Gramineae* | | Herb | |
