# Supplementary material for: Climatic Stress during Stand Development Alters the Sign and Magnitude of Age-Related Growth Responses in a Subtropical Mountain Pine
Source: PLoS One. 2015 May 14;10(5):e0126581. doi: 10.1371/journal.pone.0126581 (PMC4431836; doi:10.1371/journal.pone.0126581)
Supplement: S3 Table — (DOCX) [file pone.0126581.s007.docx]

**S3 Table. Summary statistics of ring width index chronologies of *Pinus taiwanensis* for the common interval 1975-2009.**

| Population | R_BT_ | EPS | MS | Gini | AC1 |
| --- | --- | --- | --- | --- | --- |
| Top | 0.317 | 0.926 | 0.170 | 0.101 | 0.342 |
| High | 0.283 | 0.923 | 0.183 | 0.103 | 0.231 |
| Middle | 0.123 | 0.754 | 0.220 | 0.136 | 0.361 |
| Low | 0.370 | 0.938 | 0.414 | 0.239 | 0.289 |
| Bottom | 0.286 | 0.915 | 0.390 | 0.213 | 0.264 |

R_BT_: correlation between the trees; EPS: expressed population signal; MS: mean sensitivity; Gini: Gini coefficient; AC1: 1st order autocorrelation.
