# Supplementary material for: Climatic Stress during Stand Development Alters the Sign and Magnitude of Age-Related Growth Responses in a Subtropical Mountain Pine
Source: PLoS One. 2015 May 14;10(5):e0126581. doi: 10.1371/journal.pone.0126581 (PMC4431836; doi:10.1371/journal.pone.0126581)
Supplement: S4 Table — The models were parameterized using one predictor variable (i.e. tree age, mean annual temperature or annual precipitation) and different functional forms (i.e. linear or quadratic). (DOCX) [file pone.0126581.s008.docx]

**S4 Table. Comparison of basal area increment and relative tree growth models parameterized using Akaike Information Criterion (AIC).** The models were parameterized using one predictor variable (i.e. tree age, mean annual temperature or annual precipitation) and different functional forms (i.e. linear or quadratic).

| **Basal area increment (mm^2^ yr^-1^)** | | | | | | | |
| --- | --- | --- | --- | --- | --- | --- | --- |
| **All developmental stages** | **NP** | **AIC** | **∆AIC** | **Mature stage data** | **NP** | **AIC** | **∆AIC** |
| *Tree age* |  |  |  | *Tree age* |  |  |  |
| **Quadratic** | **3** | **7068.23** | **0.00** | **Quadratic** | **3** | **6704.16** | **0.00** |
| Linear | 2 | 7273.78 | 205.54 | Linear | 2 | 7424.99 | 720.83 |
| *Mean annual temperature* | | |  | *Mean annual temperature* | |  |  |
| **Quadratic** | **3** | **6889.50** | **0.00** | Quadratic | 3 | 9727.62 | 7.69 |
| Linear | 2 | 7284.43 | 394.93 | **Linear** | **2** | **9719.93** | **0.00** |
| *Annual precipitation* | |  |  | *Annual precipitation* |  |  |  |
| Quadratic | 3 | 7256.72 | 7.76 | Quadratic | 3 | 4106.90 | 5.91 |
| **Linear** | **2** | **7248.96** | **0.00** | **Linear** | **2** | **4101.00** | **0.00** |
| **Relative tree growth (% yr^-1^)** | | | | | | | |
| **All developmental stages** | **NP** | **AIC** | **∆AIC** | **Mature stage data** | **NP** | **AIC** | **∆AIC** |
| *Tree age* |  |  |  | *Tree age* |  |  |  |
| **Quadratic** | **3** | **6704.16** | **0.00** | Quadratic | 3 | 5110.88 | 2.66 |
| Linear | 2 | 7424.99 | 356.76 | **Linear** | **2** | **5108.21** | **0.00** |
| *Mean annual temperature* | |  |  | *Mean annual temperature* | |  |  |
| Quadratic | 3 | 9727.62 | 7.69 | **Quadratic** | **3** | **4492.55** | **0.00** |
| **Linear** | **2** | **9719.93** | **0.00** | Linear | 2 | 4516.54 | 23.98 |
| *Annual precipitation* | |  |  | *Annual precipitation* |  |  |  |
| Quadratic | 3 | 11606.97 | 0.00 | Quadratic | 3 | 5105.17 | 0.00 |
| **Linear** | **2** | **11611.82** | **4.85** | **Linear** | **2** | **5110.91** | **5.74** |

The best fitting model is given in ∆AIC value in bold. NP is the number of parameters for the fixed effects.
