# Supplementary material for: Climatic Stress during Stand Development Alters the Sign and Magnitude of Age-Related Growth Responses in a Subtropical Mountain Pine
Source: PLoS One. 2015 May 14;10(5):e0126581. doi: 10.1371/journal.pone.0126581 (PMC4431836; doi:10.1371/journal.pone.0126581)
Supplement: S5 Table — We tested seven candidate models without interactions and three models that include all possible pair-wise interactions between tree age and climatic variables using Akaike Information Criterion (AIC). (DOCX) [file pone.0126581.s009.docx]

**S5 Table. Comparison of alternative basal area increment models and relative tree growth models.** We tested seven candidate models without interactions and three models that include all possible pair-wise interactions between tree age and climatic variables using Akaike Information Criterion (AIC).

| **Nested models basal area increment (mm^2^ yr^-1^), all developmental stages** | | | | | | | | | | | | | | |
| --- | --- | --- | --- | --- | --- | --- | --- | --- | --- | --- | --- | --- | --- | --- |
| **Main effects** | | | | | | **Pairwise Interactions** | | | | **NP** | | **AIC** | | **∆AIC** |
| **TA** | | **MAT** | | **PP** | | **TA × MAT** | | **TA × PP** | |  |  |  |  |  |
| ***NL*** | | ***NL*** | | ***L*** | | ***L*** | | ***-*** | | ***7*** | | ***6551.912*** | | ***0.00*** |
| NL | | NL | | L | | L | | L | | 8 | | 6557.941 | | 6.03 |
| NL | | NL | | L | | - | | - | | 6 | | 6765.975 | | 214.06 |
| NL | | NL | | L | | - | | L | | 7 | | 6769.069 | | 217.16 |
| NL | | NL | | - | | - | | - | | 5 | | 6783.86 | | 231.95 |
| - | | NL | | L | | - | | - | | 4 | | 6868.41 | | 316.50 |
| - | | NL | | - | | - | | - | | 3 | | 6889.50 | | 337.58 |
| NL | | - | | L | | - | | - | | 4 | | 7049.07 | | 497.15 |
| NL | | - | | - | | - | | - | | 3 | | 7068.234 | | 516.32 |
| - | | - | | L | | - | | - | | 2 | | 7248.96 | | 697.05 |
| - | | - | | - | | - | | - | | 1 | | 7276.74 | | 724.82 |
| **Nested models basal area increment (mm^2^ yr^-1^), mature stages** | | | | | | | | | | | | | | |
| **Main effects** | | | | | | **Pairwise Interactions*** | | | | **NP** | | **AIC** | | **∆AIC** |
| **TA** | | **MAT** | | **PP** | | **TA × MAT** | | **TA × PP** | |  |  |  |  |  |
| *-* | | *NL* | | *L* | | *-* | | *-* | | *4* | | *3920.384* | | *0.00* |
| **L** | | **NL** | | **L** | | **-** | | **-** | | **5** | | **3928.148** | | **7.76** |
| L | | NL | | L | | L | | - | | 6 | | 3935.329 | | 14.95 |
| L | | NL | | L | | - | | L | | 6 | | 3935.738 | | 15.35 |
| - | | NL | | - | | - | | - | | 3 | | 3936.218 | | 15.83 |
| L | | NL | | L | | L | | L | | 7 | | 3942.969 | | 22.59 |
| L | | NL | | - | | - | | - | | 4 | | 3943.981 | | 23.60 |
| - | | - | | L | | - | | - | | 2 | | 4100.999 | | 180.62 |
| - | | - | | - | | - | | - | | 1 | | 4104.135 | | 183.75 |
| L | | - | | L | | - | | - | | 3 | | 4108.489 | | 188.11 |
| L | | - | | - | | - | | - | | 2 | | 4111.631 | | 191.25 |
| **Nested models relative tree growth (% yr^-1^), all developmental stages** | | | | | | | | | | | | | | |
| **Main effect*** | | | | | **Pairwise Interactions** | | | | **NP** | | **AIC** | | **∆AIC** | |
| **TA** | **MAT** | | **PP** | | **TA × MAT** | | **TA × PP** | |  |  |  |  |  |  |
| ***NL*** | ***NL*** | | ***L*** | | ***L*** | | ***L*** | | ***8*** | | ***6639.045*** | | ***0.00*** | |
| NL | NL | | L | | - | | L | | 7 | | 6654.46 | | 15.42 | |
| NL | NL | | L | | L | | - | | 7 | | 6666.64 | | 27.60 | |
| NL | NL | | L | | - | | - | | 6 | | 6685.19 | | 46.15 | |
| NL | NL | | - | | - | | - | | 5 | | 6696.59 | | 57.55 | |
| NL | - | | L | | - | | - | | 4 | | 6703.21 | | 64.17 | |
| NL | - | | - | | - | | - | | 3 | | 6704.16 | | 65.12 | |
| - | NL | | - | | - | | - | | 3 | | 9727.62 | | 3088.58 | |
| - | NL | | L | | - | | - | | 4 | | 9735.89 | | 3096.84 | |
| - | - | | L | | - | | - | | 2 | | 11611.82 | | 4972.78 | |
| - | - | | - | | - | | - | | 1 | | 11668.14 | | 5029.10 | |
| **Nested models relative tree growth (% yr^-1^), mature stages** | | | | | | | | | | | | | | |
| **Main effects** | | | | | **Pairwise Interactions** | | | | **NP** | | **AIC** | | **∆AIC** | |
| **TA** | **MAT** | | **PP** | | **TA × MAT** | | **TA × PP** | |  |  |  |  |  |  |
| *-* | *NL* | | *L* | | *-* | | *-* | | *4* | | *4453.845* | | *0.00* | |
| **L** | **NL** | | **L** | | **-** | | **-** | | **5** | | **4458.112** | | **4.27** | |
| L | NL | | L | | *-* | | L | | 6 | | 4464.40 | | 10.56 | |
| L | NL | | L | | L | | - | | 6 | | 4465.856 | | 12.01 | |
| L | NL | | L | | L | | L | | 7 | | 4472.164 | | 18.32 | |
| - | NL | | - | | - | | - | | 3 | | 4492.553 | | 38.71 | |
| L | NL | | - | | - | | - | | 4 | | 4496.841 | | 43.00 | |
| - | - | | L | | - | | - | | 5 | | 5110.91 | | 657.07 | |
| L | - | | L | | - | | - | | 3 | | 5111.643 | | 657.80 | |
| - | - | | - | | - | | - | | 1 | | 5107.415 | | 653.57 | |
| L | - | | - | | - | | - | | 2 | | 5108.213 | | 654.37 | |

Variables were included as “NL” (non-linear form), “L” (linear form) or “–” (not included). We used all possible combinations of the three *RTG* predictors selected (main effects and pairwise interactions with tree age): tree age (*TA*), mean annual temperature (*MAT*) and annual precipitation (*PP*). NP is the number of parameters for the fixed effects. The best fitting model is determined by ∆AIC value of zero and it is given in italics. The selected model includes the effect of tree age and it is given in bold.
